# Conjugates of Chitosan and Calcium Alginate with Oligoproline and Oligohydroxyproline Derivatives for Potential Use in Regenerative Medicine

**DOI:** 10.3390/ma13143079

**Published:** 2020-07-10

**Authors:** Joanna Wasko, Justyna Fraczyk, Angelika Becht, Zbigniew J. Kaminski, Sandra Flinčec Grgac, Anita Tarbuk, Marta Kaminska, Mariusz Dudek, Eulalia Gliscinska, Zbigniew Draczynski, Beata Kolesinska

**Affiliations:** 1 Institute of Organic Chemistry, Faculty of Chemistry, Lodz University of Technology, Zeromskiego 116, 90-924 Lodz, Poland; joanna.wasko@edu.p.lodz.pl (J.W.); justyna.fraczyk@p.lodz.pl (J.F.); angelika.becht@dokt.p.lodz.pl (A.B.); zbigniew.kaminski@p.lodz.pl (Z.J.K.); 2 Department of Textile Chemistry and Ecology, University of Zagreb Faculty of Textile Technology, Prilaz baruna Filipovića 28a, HR-10000 Zagreb, Croatia; sflincec@ttf.hr (S.F.G.); anita.tarbuk@ttf.hr (A.T.); 3 Institute of Materials Science and Engineering, Faculty of Mechanical Engineering, Lodz University of Technology, Stefanowskiego 1/15, 90-924 Lodz, Poland; marta.kaminska@p.lodz.pl (M.K.); mariusz.dudek@p.lodz.pl (M.D.); 4 Institute of Material Sciences of Textiles and Polymer Composites, Faculty of Material Technologies and Textile Design, Lodz University of Technology, Zeromskiego 116, 90-924 Lodz, Poland; eulalia.klata@p.lodz.pl (E.G.); zbigniew.draczynski@p.lodz.pl (Z.D.)

**Keywords:** conjugate of polysaccharides with peptides, alginate, chitosan, proline, hydroxyproline derivatives, mimetic of functional collagen fragments, stable spatial structure

## Abstract

New materials that are as similar as possible in terms of structure and biology to the extracellular matrix (external environment) of cells are of great interest for regenerative medicine. Oligoproline and oligohydroxyproline derivatives (peptides **2**–**5**) are potential mimetics of collagen fragments. Peptides **2**–**5** have been shown to be similar to the model collagen fragment (H-Gly-Hyp-Pro-Ala-Hyp-Pro-OH, **1**) in terms of both their spatial structure and biological activity. In this study, peptides **2**–**5** were covalently bound to nonwovens based on chitosan and calcium alginate. Incorporation of the peptides was confirmed by Fourier transform -infrared (FT-IR) and zeta potential measurements. Biological studies (cell metabolic activity by using 3-(4,5-dimethylthiazol-2-yl)-2,5-diphenyltetrazolium bromide (MTT) test and Live/Dead assay) proved that the obtained peptide-polysaccharide conjugates were not toxic to the endothelial cell line EA.hy 926. In many cases, the conjugates had a highly affirmative influence on cell proliferation. The results of this study show that conjugates of chitosan and calcium alginate with oligoproline and oligohydroxyproline derivatives have potential for use in regenerative medicine.

## 1. Introduction

The aim of biomimetics of the cell environment is to constitute a matrix for cell deposition that affects cells in such a way that they are able to function as if they were in their natural environment [1]. Thus, materials used as cell scaffolds must provide an appropriate cell microenvironment for adequate tissue growth [2,3]. In addition, biomaterials must ensure cell adhesion, proliferation, differentiation and finally tissue regeneration. The chemical composition, physical properties and biological activity of a scaffold are therefore its key characteristics and only a balanced combination of all them can guarantee tissue regeneration. A variety of materials, both natural and synthetic, have been used for tissue regeneration [4,5,6]. Synthetic biodegradable compounds belonging to the group of polyesters and co-polyesters have found the widest applications [7,8,9,10]. Synthetic scaffolds characterized by biodegradability and the possibility of modulating their mechanical and physical properties have been developed, with structural parameters adapted to the needs of various types of tissues. The problem with this group of compounds is that they can cause acidification of the environment, resulting from the degradation of polyesters to the corresponding mers. This leads to inflammatory processes at the implantation site. Hybrid scaffolds, resulting from the merger of synthetic and natural materials, have also proven be useful in regenerative medicine. Their formation involves the modification of surface of synthetic polymers using biomolecules found in extracellular matrix (ECM) such as fibrinogen or collagen. This improves their properties and biocompatibility, combining the advantages of both types of structure—the mechanical strength of synthetic polymers and the ability of natural compounds to interact with cells [11,12].

Currently, research underway to develop new materials based solely on natural polymers, including polysaccharides and proteins, especially those that are part of the extracellular matrix. These natural materials are ideally suited to the function of scaffolds in regenerative medicine. They include proteins that are able to undergo self-assembly and form nanofiber structures. Collagens are particularly useful, including collagens II, III, V and XI and in particular type I and type VII collagens represent the most fundamental components in the extracellular matrix and play crucial role in wound healing [13,14,15,16,17,18,19,20,21]. However, the main disadvantage of collagen is its animal origin (xenogenic material) or allogenic origin, which activates the immune system and may lead to its rejection. In addition, collagen of xenogenic origin may not be sufficiently clean, which risks the effects of unknown impurities on the regeneration process [22]. The additional purification of collagen used in transplants results in increased costs compared to their synthetic counterparts [23].

The use of protein-based scaffolds is almost impossible, due to the poor mechanical properties of proteins. Proteins undergo enzymatic degradation and must therefore be administered continuously. Therefore, proteins are usually deposited on materials that provide mechanical durability. However, due to their random orientation on the cell surface, only some of the proteins have the proper placement for adhesion [24]. The surface structure of the material, characterized by the load, wettability and topography, can affect the spatial arrangement of deposited proteins [25,26,27,28,29,30,31,32]. On hydrophobic surfaces, deposited proteins will strive to increase their interactions with the hydrophobic substituents of hydrophobic amino acids, which may result in protein denaturation and a different way of presenting the protein motifs responsible for cell adhesion [33,34]. Most of these problems can be eliminated by using small peptides immobilized on materials as presenting cell recognition motifs. Peptides are characterized by higher stability under sterilization conditions and at elevated temperatures. They are also less sensitive to changes in pH [35] and storage conditions [36]. Due to the small area (volume) of peptides, it is possible to achieve a higher degree of peptide filling on the surface of the modified material. This, in turn, makes it possible to compensate for the lower activity of peptides compared to proteins, with respect to cell adhesion [37]. The extracellular matrix (ECM) consists of proteins containing numerous structural motifs determining cell adhesion, while a single peptide is also a single motif of cell adhesion. Thus, it is possible to achieve selectivity for the required cell type, which translates into selectivity of action on one type of receptor. Both linear and cyclic peptides can be a starting point for the design and synthesis of peptidomimetics [38]. Quite often, linear peptides have a low degree of enzymatic degradation (even in vivo) [39]. Cyclic peptides [40,41,42] are characterized by stability to enzymatic degradation and stability over time.

Collagen is characterized by structural motif containing three parallel left-handed oligopeptide chains with a helical PPII conformation drape around each other to form a right-handed triple helix [43,44]. This is the main protein component of the extracellular matrix and at the same time is a frequent structural motif of protein-derived scaffolds for regenerative medicine. In collagen, folding PPII helices into a triple helix forces every third rest to be glycine, giving the recurring Xaa-Yaa-Gly motif [45]. The Xaa and Yaa residues of collagen are usually L-proline (ca. 30%) and L-hydroxyproline (ca. 40%). The fragment Pro-Hyp-Gly is the most frequent triplet in collagen [46]. Helical PPII conformation is characteristic for oligoproline derivatives and proline-rich peptides/proteins. The conformational stability of such peptides means that they have been classified as one of the groups of cell-penetrating peptides that can be utilized as a drug delivery system [47].

In this study, we investigate whether oligoproline and oligohydroxyproline derivatives can be used as conformationally stable collagen fragment mimetics in regenerative medicine. We also examine whether the covalent attachment of oligoproline derivatives to polysaccharides allows for the improvement of conjugate activity compared to the individual components. Chitosan and calcium alginate were selected as polysaccharide matrices based on their widely proven utility in medicine, including regenerative medicine [48,49,50].

## 2. Materials and Methods

### 2.1. Synthesis and Characterization of Peptides **1**–**5**

Model collagen fragments H-Gly-Hyp-Pro-Ala-Hyp-Pro-OH (**1**), H-(Pro)_6_-OH (**2**), H-(Pro)_9_-OH (**3**), H-(Hyp)_6_-OH (**4**) and H-(Hyp)_9_-OH (**5**) were synthesized on chlorotrityl resin according the Fmoc/tBu strategy.

Analytical RP-HPLC was performed on a LC Dionex UltiMate 3000 (ThermoFisher Scientific, Waltham, MA, USA), using a Kinetex Reversed Phase C18 column (100 × 4.6 mm). Gradients of 0.1% TFA in H_2_O (B) and 0.1% TFA in CH_3_CN (A) were used, at a flow rate 0.4 mL/min with UV detection at 220 and 254 nm. Preparative HPLC was done on a CombiFlash, EZPrep, Teledyne ISCO (Lincoln, NE, USA) using a Supelco Discovery BIO Wide Pore C18 column (25 cm × 21.2 mm, 10 mm; Sigma-Aldrich) flow rate 5 mL/min, detection wavelengths 220 and 254 nm, gradient ratio A (0.1% TFA in CH_3_CN) and B (0.1% TFA in H_2_O) 0:100 to 18:82 in 30 min, followed by an isocratic run for 5 min.

Mass Spectrometry analysis was performed on a Bruker microOTOF-QIII (Bruker Corporation, Billerica, MA, USA).

#### 2.1.1. General Procedures and Synthesis of Peptides **1**–**5**

Attachment of C-terminal amino acid on 2-chlorotrityl chloride resin (GP1).

2-chlorotrityl chloride resin with an initial load of 1.0 mmol/g (1 g corresponds to 1 equivalent (1 mmol)) was used for the reaction. The Fmoc-protected amino acid (3 equivalent related to the resin) and 6 equivalent of EtNiPr_2_ were dissolved in CH_2_Cl_2_ (10 mL/g resin) and added to 2-chlorotrityl chloride resin, which had previously undergone a swelling process in CH_2_Cl_2_ for 1 h. The suspension was shaken for 60 min. The polymer was filtered and washed with CH_2_Cl_2_/MeOH/EtNiPr_2_ 17:2:1 (3 ×), DMF (2 ×) and CH_2_Cl_2_ (3 ×). The modified resin was dried in a vacuum desiccator to constant mass.

Typical Coupling Procedure (GP 2).

For the coupling procedure, 3 equivalent of the amino acid, 3 equivalent of 4-(4,6-dimethoxy-1,3,5-triazin-2-yl)-4-methylmorpholinium toluene-4-sulfonate (DMT/NMM/TosO^−^) [51] and 6 equivalent of N-methylmorpholine (NMM) were dissolved in N,N-dimethylformamide (DMF) (final amino acid concentration in DMF 0.5 mM). The solution was added to the resin. The suspension was shaken for 1–2 h. The chloranil test was used to monitor the completion of the reaction.

Deprotection (GP 3).

The Fmoc protecting group was removed using 25% piperidine solution in DMF (2 × 5 min).

Cleavage from the Resin (GP 4).

The peptides were cleaved from the resin using a mixture (ca. 2 mL/0.1 g resin) of 95% TFA (2,2,2-trifluoroacetic acid), 2.5% H_2_O and 2.5% TIS (triisopropylsilane). Cleavage was performed over 4 h at room temperature. The crude product was lyophilized, identified by MS. The purity of final product was determined by RP-HPLC.

**Figure 1:**
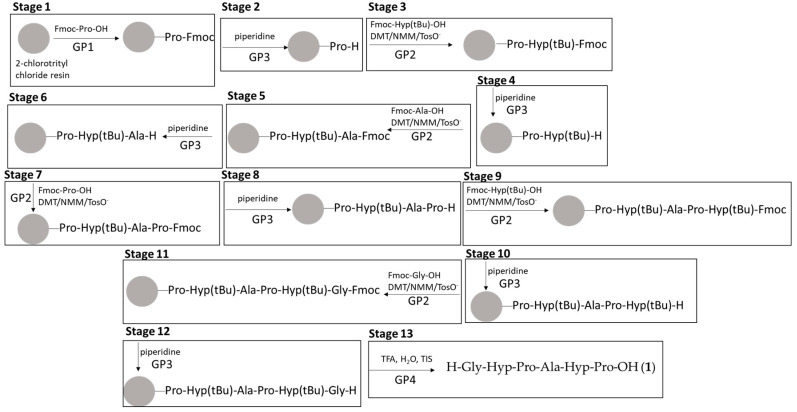
Block diagram of peptide **1** synthesis.

A block diagram of peptide synthesis, for example peptide **1**, is shown in Figure 1.

Synthesis of H-Gly-Hyp-Pro-Ala-Hyp-Pro-OH (**1**)

Starting materials for each coupling—resin (1.0 g, 1.0 mmol), Fmoc-Pro-OH (1.012 g, 3.0 mmol), Fmoc-Ala-OH (0.934 g, 3.0 mmol), Fmoc-Hyp(tBu)-OH (1.228 g, 3.0 mmol), Fmoc-Gly-OH (0.892 g, 3.0 mmol). DMT/NMM/TosO^−^ (1.239 g, 3.0 mmol) and NMM (0.66 mL, 6.0 mmol). The peptide was cleaved from the resin according to the procedure GP 4.

High Performance Liquid Chromatography (HPLC) (15–95% A in 30 min): *t*_R_ 8.22 min, purity = 98.95%.

Liquid Chromatography Mass Spectrometry (LC/MS): 567.6223 ([M + H]^+^, C_25_H_39_N_6_O_9_^+^; calc. 567.60).

Synthesis of H-(Pro)_6_-OH (**2**)

Starting materials for each coupling—resin (1.0 g, 1.0 mmol), Fmoc-Pro-OH (1.012 g, 3.0 mmol), DMT/NMM/TosO^−^ (1.239 g, 3.0 mmol) and NMM (0.66 mL, 6.0 mmol). The peptide was cleaved from the resin according to the procedure GP 4.

HPLC (15–95% A in 30 min): *t*_R_ 14.56 min, purity = 99.15%.

LC/MS: 601.7064 ([M+H]^+^, C_30_H_45_N_6_O_7_^+^; calc. 601.32).

Synthesis of H-(Pro)_9_-OH (**3**)

Starting materials for each coupling—resin (1.0 g, 1.0 mmol), Fmoc-Pro-OH (1.012 g, 3.0 mmol), DMT/NMM/TosO^−^ (1.239 g, 3.0 mmol) and NMM (0.66 mL, 6.0 mmol). The peptide was cleaved from the resin according to the procedure GP 4.

HPLC (15–95% A in 30 min): *t*_R_ 15.71 min, purity = 99.25%.

LC/MS: 892.4854 ([M+H]^+^, C_45_H_66_N_9_O_10_^+^; calc. 892.05).

Synthesis of H-(Hyp)_6_-OH (**4**)

Starting materials for each coupling—resin (1.0 g, 1.0 mmol), Fmoc-Hyp(tBu)Pro-OH (1.228 g, 3.0 mmol), DMT/NMM/TosO^−^ (1.239 g, 3.0 mmol) and NMM (0.66 mL, 6.0 mmol). The peptide was cleaved from the resin according to the procedure GP 4.

HPLC (15–95% A in 30 min): *t*_R_ 11.71 min, purity = 98.95%.

LC/MS: 697.6966 ([M + H]^+^, C_30_H_45_N_6_O_13_^+^; calc. 697.70).

Synthesis of H-(Hyp)_9_-OH (**5**)

Starting materials for each coupling—resin (1.0 g, 1.0 mmol), Fmoc-Hyp(tBu)-OH (1.228 g, 3.0 mmol), DMT/NMM/TosO^−^ (1.239 g, 3.0 mmol) and NMM (0.66 mL, 6.0 mmol). The peptide was cleaved from the resin according to the procedure GP 4.

HPLC (15–95% A in 30 min): *t*_R_ 13.88 min, purity = 98.90%.

LC/MS: 1036.4396 ([M + H]^+^, C_45_H_66_N_9_O_19_^+^; calc. 1036.05).

#### 2.1.2. CD Studies

CD studies were done with a Jasco J–1500 spectrometer Far-UV (ABLE JASCO Polska, Cracow, Poland) and rectangular quartz cuvette (1 mm path length, Hellma). The samples were prepared in water (LC-MS grade water, Merck Warsaw, Poland) and methanol (LC-MS, Merck Polska, Warsaw, Poland), at a concentration of 0.1 mg/mL. All studies were carried out at room temperature. CD spectra were measured in the range of 190–270 nm. The experimental parameters—data pitch, 5 nm; scanning mode, continuous; scanning speed, 100 nm/min; bandwidth, 3 nm; integration time, 1 s.

#### 2.1.3. Raman Spectroscopy Studies

Peptides **1**–**5** were analyzed on a Renishaw inVia Raman Microscope equipped with a 532 nm laser arranged in a backscattering geometry, wavenumber range 100–3200 cm^−1^. All measurements were carried out at room temperature in an ambient air atmosphere.

### 2.2. Preparation of Nonwovens

Calcium alginate fibers were made by the wet solution method at the Institute of Material Sciences of Textiles and Polymer Composites of the Lodz University of Technology. Spinning conditions were in accordance with the procedure described by Bogun et al. [52]. Calcium alginate fibers were obtained from sodium alginate Protanal LF 10/60LS (FMC Biopolymer, Sandvika, Norway) using a calcium chloride solution as a coagulation solvent.

Chitosan fibers were also obtained by wet-spinning from a solution according to the adapted procedure presented by Wawro and Pighinelli [53]. Commercial chitosan from Primex Co., Myre, Norway, degree of deacetylation 90% (Fourier transform infrared (FTIR)), Mv = 350 kDa was used to form the fibers.

To manufacture needle-punched nonwovens, a fleece with a cross-system arrangement of fiber was obtained on a Befama (BEFAMA Sp. z o.o., Kalina, Poland) laboratory roller carding machine with an elastic covering, equipped with an Asselin (ANDRITZ Asselin-Thibeau S.A.S., Elbeuf sur Seine, France) horizontal stacker. Needle punching of the fleece was carried out on an Asseline needle punching machine (ANDRITZ Asselin-Thibeau S.A.S., Elbeuf sur Seine, France) with an upper needle plate. The technological parameters of needle punching were as follows—type of needles—15 × 18 × 40 × 3 ½ RB (Groz-Beckert^®^, Albstadt-Ebingen, Germany); needle density 9.5; number of needle punches 60/cm^2^; depth of needle punching 12 mm.

The mass per square meter of nonwovens was determined according to the ISO 9073-1:1989 standard at 70 g/m^2^ for calcium alginate nonwoven and 90 g/m^2^ for chitosan nonwoven.

Characteristic of prepared chitosan nonwoven—the thickness—2.2 mm, the tensile strength in the longitudinal direction—19.3 N, the tensile strength in the transverse direction—15.3 N, the elongation in the longitudinal direction—88.8%, the elongation in the transverse direction—61.5%.

Characteristic of prepared calcium alginate nonwoven—the thickness—2.0 mm, the tensile strength in the longitudinal direction—5.7 N, the tensile strength in the transverse direction—11.6 N, the elongation in the longitudinal direction—74.1%, the elongation in the transverse direction—50.0%.

Determination of nonwovens thickness was measured according to the ISO 9073-2:1995 standard.

Determination of tensile strength and elongation was carried out according to the ISO 29073-3:1989 standard.

### 2.3. Synthesis of Polysaccharide-Peptide Conjugates

#### 2.3.1. Synthesis of conjugates of chitosan with peptides **2**–**5**

Details on the preparation of conjugates of chitosan with peptides **2**–**5** are given in Table 1.

A 5 cm × 8 cm chitosan nonwoven (0.36 g, which corresponds to 2 mmol of free amino groups of D-glucosamine units) was used for the reaction. In all cases, an equal amount of polysaccharide nonwoven was used. Before the coupling reaction, the chitosan nonwoven was treated with a 25% aqueous solution (v:v) of N-methylmorpholine (NMM) (50 mL) for 5 min, then washed with H_2_O (50 mL), a mixture of water and THF (1:1, v:v) ( 50 mL) and again with THF (50 mL) (stage 1).

Peptide preactivation (stage 2) was performed in parallel. Equimolar amounts of the peptide, DMT/NMM/TosO^−^ and 3 equivalent of NMM were used. Preactivation was carried out in a mixture of DMF and water (7:3, v:v) (20 mL) for 5 min, with vigorous stirring.

Preactivation of peptide **2**: H-(Pro)_6_-OH (0.120 g, 0.2 mmol), DMT/NMM/TosO^−^ (82.6 mg, 0.2 mmol), NMM (66 μL, 0.6 mmol).

Preactivation of peptide **3**: H-(Pro)_9_-OH (0.179 g, 0.2 mmol), DMT/NMM/TosO^−^ (82.6 mg, 0.2 mmol), NMM (66 μL, 0.6 mmol).

Preactivation of peptide **4**: H-(Hyp)_6_-OH (0.140 g, 0.2 mmol), DMT/NMM/TosO^−^ (82.6 mg, 0.2 mmol), NMM (66 μL, 0.6 mmol).

Preactivation of peptide **5**: H-(Hyp)_9_-OH (0.207 g, 0.2 mmol), DMT/NMM/TosO^−^ (82.6 mg, 0.2 mmol), NMM (66 μL, 0.6 mmol).

The chitosan nonwovens were treated with solutions of activated **2**–**5** peptides (four separate reactions), at room temperature, with gentle shaking, reaction time 24 h (stage 3). After this time, the solutions were removed and the chitosan nonwovens were washed successively with a DMF-H_2_O mixture (1:1, 50 mL), DMF (50 mL), H_2_O (50 mL), THF (2 × 50 mL) and EtOH (50 mL). They were dried in a vacuum desiccator to constant weight.

#### 2.3.2. Synthesis of Conjugates of Calcium Alginate with Peptides **2**–**5**

Details on the preparation of conjugates of calcium alginate with peptides **2**–**5** are given in Table 2.

Calcium alginate nonwoven was used for the reaction, with dimensions of 5 cm × 8 cm (0.28 g, corresponding to an average of 1.6 mmol of carboxylate groups and 1.6 mmol of free hydroxyl groups of the composite unit from MM, GG or MG/GM). In all cases, an equal amount of polysaccharide matrix was used.

There was no separate preactivation process. Immediately after all reagents had been dissolved in the mixture of DMF and H_2_O (7:3, v:v) (20 mL), the solutions were poured onto calcium alginate nonwovens.

Reaction with peptide **2**: H-(Pro)_6_-OH (0.192 g, 0.32 mmol), DMT/NMM/TosO^−^ (0.396 g, 0.96 mmol), NMM (123 μL, 1.12 mmol).

Reaction with peptide **3**: H-(Pro)_9_-OH (0.286 g, 0.32 mmol), DMT/NMM/TosO^−^ (0.396 g, 0.96 mmol), NMM (123 μL, 1.12 mmol).

Reaction with peptide **4**: H-(Hyp)_6_-OH (0.224 g, 0.32 mmol), DMT/NMM/TosO^−^ (0.396 g, 0.96 mmol), NMM (123 μL, 1.12 mmol).

Reaction with peptide **5**: H-(Hyp)_9_-OH (0.331 g, 0.32 mmol), DMT/NMM/TosO^−^ (0.396 g, 0.96 mmol), NMM (123 μL, 1.12 mmol).

The calcium alginate nonwovens were treated with solutions of peptides **2**–**5** (four separate reactions), at room temperature, with gentle shaking, reaction time 24 h. After this time, the solutions were removed. The nonwovens were washed successively with a mixture of DMF and H_2_O (1:1, v:v, 50 mL), DMF (50 mL), H_2_O (50 mL), THF (2 × 50 mL) and EtOH (50 mL), dried in a vacuum desiccator to constant weight.

### 2.4. Characteristic of Polysaccharide-Peptide Conjugates

#### 2.4.1. FT-IR Studies

The samples were analyzed using attenuated total reflectance (ATR) Fourier transform infrared (FT-IR) spectroscopy (Perkin Elmer, software Spectrum 100, PerkinElmer, Inc. Waltham, MA, United States). Four scans were performed for each sample, at a resolution of 4 cm^−1^ between 4000 cm^−1^ and 380 cm^−1^.

#### 2.4.2. Electrokinetic Potential Studies

**Figure 2:**
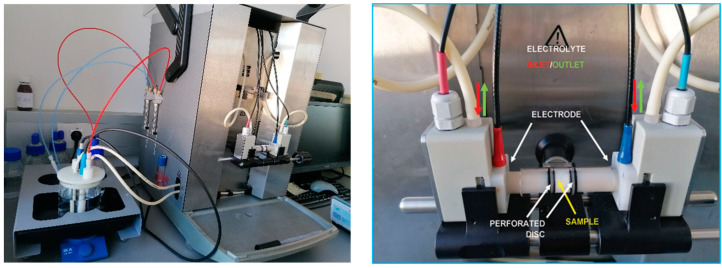
Test stand for determining the zeta potential.

Electrokinetic potential (zeta, ζ) was calculated using the Helmholtz-Smoluchowski equation [54] after measuring streaming potential on a SurPASS (Anton Paar GmbH, Graz, Austria) [55], using the cylindrical cell, in the pH range of 3.5–10 and 0.001 M KCl as the electrolyte. Four measurements were performed for each sample. The Isoelectric Point (IEP) was calculated. The zeta potential was measured on samples (0.4 g) of polysaccharide nonwovens modified with peptides **2**–**5**. Figure 2 shows the principle of measurement in the cylindrical cell of the SurPASS electrokinetic analyzer.

### 2.5. Examination of Cell Cytotoxicity

The endothelial cell line EA.hy 926 (ATCC, Manassas, VA, USA) [56] was used. The MTT test was performed using endothelial cells of the EA.hy 926 line. The extracts (2 mg/mL) were incubated in DMEM (Corning) for 48 h at 37 °C. Cells were seeded into a 96-well plate with 1 × 10^4^ cells per well and grown under standard conditions (37 °C, 5% CO_2_ in humid air). The next day, the medium was replaced with extracts. After 24 h of treatment, the culture medium was removed and replaced with the MTT solution (1 mg/mL, Sigma Aldrich, Poznan, Poland) and incubated for 2 h in an incubator. The MTT solution was then decanted and 100 µL of isopropanol (Sigma Aldrich, Poznan, Poland) was added to each well. Absorbance was measured at 570 nm and 650 nm for the reference using a microplate reader (Victor X4 Perkin Elmer, PerkinElmer, Inc. Waltham, MA, USA). Cells cultured for 48 h in DMEM were used as a negative control. As a positive control were used cells incubated for 24 h, followed by the addition of DMSO, which were then cultured for another 24 h. Three samples of each type were tested and the results were presented as a percentage of the negative control with the standard deviation. Statistical significance was assessed using one-way ANOVA analysis of variance. Values for which the value **p < 0.001, ** *p* < 0.01, *p < 0.05 were considered statistically significant.

In addition, the cell viability of selected samples was assessed, using a Live/Dead Viability/Cytotoxicity Kit (Molecular Probes, Thermo Fisher Scientific, Waltham, MA, USA) containing Calcein AM, a green color for live cells and ethidium homodimer-1 (EthD-1, a red color for dead cells. The study also used EA.hy 926. The cells were cultured in a Dulbecco’s Modified Eagle’s Medium containing L–glutamine (4 mM), glucose (4.5 g/L), 15% fetal bovine serum (Biological Industries, Kibbutz Beit HaEmek, Israel) and 0.5% mixtures of streptomycin sulfate and penicillin G (Pen: 10.000 U mL^−1^; Strep: 10 mgmL^−1^; Biological Industries) under standard conditions (37 °C, in a humidified atmosphere of 5% CO_2_ in air). The samples were then washed twice with phosphate-buffered saline (PBS) and incubated with dyes (0.6 μM Calcein AM1 and 1.5 μM EthD-1) for 30 min at 37 °C. The stain solutions were removed by washing with PBS. Microscopic examinations were performed on a fluorescence microscope (Olympus GX71, Olympus, Tokyo, Japan).

## 3. Results and Discussion

Synthesis of all peptides, H-Gly-Hyp-Pro-Ala-Hyp-Pro-OH (**1**), H-(Pro)_6_-OH (Pro_6_, **2**), H-(Pro)_9_-OH (Pro_9_, **3**), H-(Hyp)_6_-OH (Hyp_6_, **4**), H-(Hyp)_9_-OH (Hyp_9_, **5**), was carried out on solid phase with 4-(4,6-dimethoxy-1,3,5-triazin-2-yl)-4-methylmorpholinium toluene-4-sulfonate (DMT/NMM/TosO^−^) as a coupling reagent [51]. The purity of the final peptides was in all cases above 98.5%.

**Figure 3:**
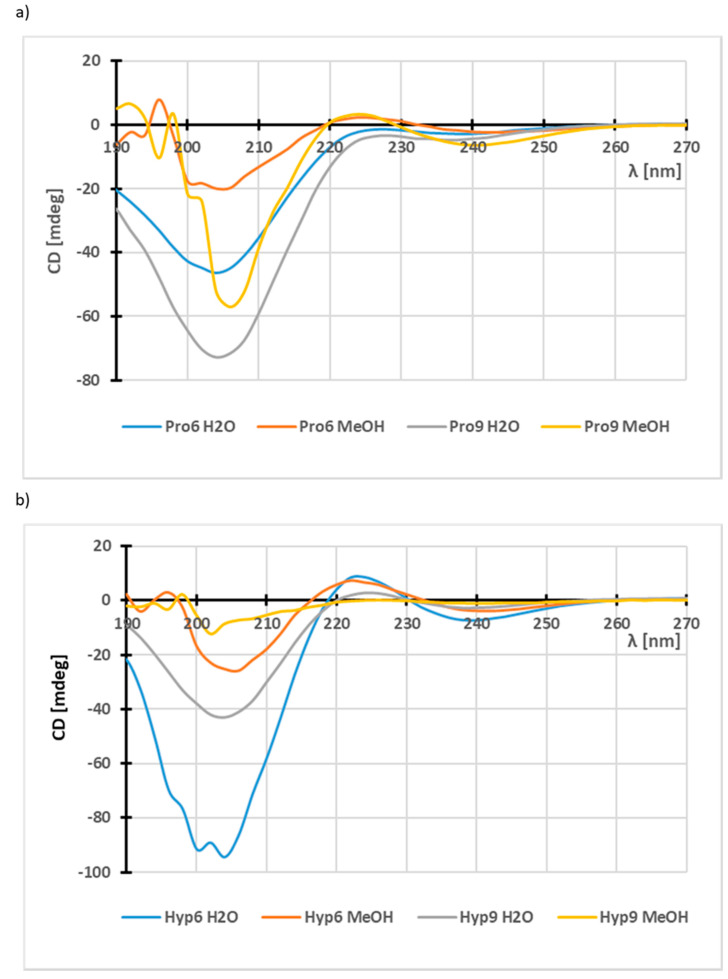
Circular dichroism (CD) spectra of oligoprolines **2**, **3** derivatives (panel (**a**)) and oligohydroxyprolines **4**, **5** (panel (**b**)).

In the first stage, we examined whether all the synthesized derivatives formed ordered spatial structures. Previous research on oligoproline derivatives had shown that even dipeptides assembled from α-Pro, β^2^h-Pro and β^3^h-Pro form stable spatial structures [57]. Hexa– and nona–peptides composed of proline and hydroxyproline were used in conformational studies. Based on CD studies in water and methanol, it was found that all the derivatives formed ordered spatial structures, which due to the similarity of the CD spectra (Figure 3) can be classified as peptides forming the right-handed PPI and the left-handed PPII helix [58,59]. The PPII helix is formed in H_2_O and organic acids [60,61], while the PPI helix dominates in hydrophobic solvents [62,63]. As a recurring Xaa-Yaa-Gly motif is characteristic for collagen, where Xaa and Yaa are usually L-proline (ca. 30%) and L-hydroxyproline (ca. 40%), it can be supposed that oligoproline and oligohydroxyproline derivatives could be structural collagen mimetics, able to form ordered spatial structures even for short peptides. CD studies of both peptides **2** and **3** composed of L-proline residues and peptides **4**, **5** composed of L-hydroxyproline residues, showed that these peptides form ordered spatial structures (Figure 3).

Diversifying the molar ellipticity for peptides **2**–**5** as a function of the amounts of amino acid residues yielded surprising results. In the case of oligoproline derivatives (peptides **2** and **3**) this relationship is intuitive, with larger numbers of residues in the peptide chain corresponding to a higher value for Pro_9_ (peptide **3**) compared to Pro_6_ peptide (peptide **2**), in methanol and in water (Figure 3a). However, there is an inverse relationship for peptides composed of L-hydroxyproline residues (Figure 3b). Hyp_6_ has higher molar ellipticity compared to peptide **5** consisting of nine L-hydroxyproline residues.

**Figure 4:**
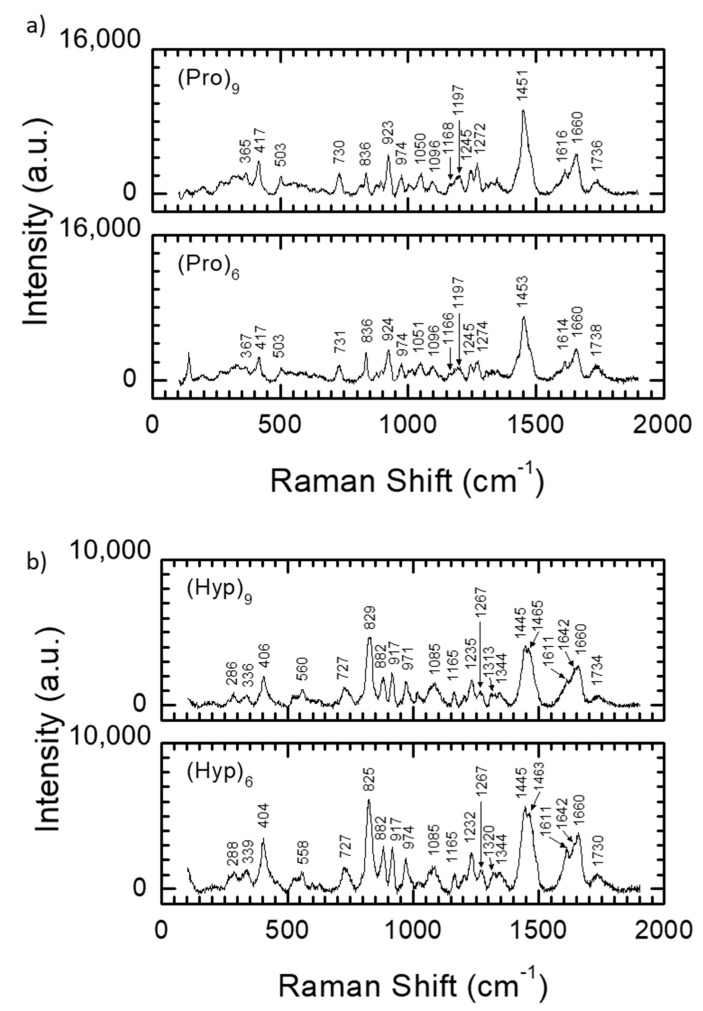
Raman spectra for peptides **2**–**5** limited to the range of 100–2000 cm^−1^: oligoprolines (**a**) and oligohydroxyprolines (**b**).

Raman spectroscopy studies [64] also confirmed that both oligoprolines and oligohydroxyprolines (Figure 4) form ordered spatial structures. Peptides **1**–**5** were placed on glass slides and examined using a Renishaw inVia Raman microscope equipped with a 532 nm laser set up in the backscattering geometry. Measurements were made in the wavelength range from 100 to 3200 cm^−1^. The structures of the peptides were studied using Raman spectroscopy. In Raman spectroscopy, three regions are characteristic for amide/peptide bonds—near 1650 cm^−1^, in 80% of cases assigned to C=O stretch (Amide I band); near 1550 cm^−1^, in 60% of cases corresponding to N-H bend and in 40% to C-N stretch (Amide II band) and near 1300 cm^−1^, corresponding in 40% of cases to C-N stretch and 30% to N-H bend (Amide III band). Figure 4 shows the Raman spectra of oligoprolines **2** and **3** (Figure 4a) and oligohydroxyprolines **4** and **5** (Figure 4b) peptides limited to the range of 100–2000 cm^−1^, where the characteristic bands for peptides occurs. The positions of the bands attributed to amide I and amide III confirm that both types of synthesized peptides have α-helix structures. Regardless of the length of the peptide chain, for both types of synthesized peptides, the Raman spectra look very similar. Detailed analysis of the positions of the bands (Table 3 and Table 4) reveals that the differences do not exceed 3 cm^−1^. The main difference between the spectra obtained for oligoprolines and oligohydroxyprolines concerns the intensity of two bands relative to others in the spectrum. In the case the structure of oligoprolines, the band at ~1451 cm^−1^ attributed to the CH_2_ scissor or CH_2_ bend vibration are more intense relative to other bands. For oligohydroxyprolines, the bands at ~825 cm^−1^ attributed to C-C stretch or CH_2_ rock vibration are much more intense than the others.

Detailed descriptions of band marks on the spectrum are presented in Table 3 and Table 4 for oligoprolines and oligohydroxyprolins, respectively.

**Figure 5:**
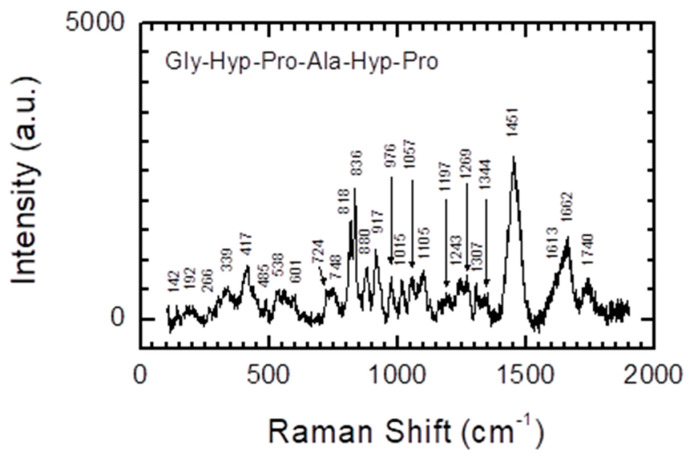
Raman spectra of H-Gly-Hyp-Pro-Ala-Hyp-Pro-OH (peptide **1**).

Raman spectroscopy was also used to evaluate the spatial structure of the model collagen fragment H-Gly-Hyp-Pro-Ala-Hyp-Pro-OH (**1**) (Figure 5, Table 5). Position bands attributed to amide I (1662 cm^−1^) and amide III (1269 cm^−1^) correspond to a predominantly α-helical structure, indicating this model peptide to be a fragment of collagen. Detailed analysis of the spectrum enables the identification of individual components of the peptide, such as proline (1740, 1613, 1451, 1344, 1243, 1197, 1057, 976, 880 and 818 cm^−1^), hydroxyproline (1740, 1613, 1344, 1243, 976, 880 and 818 cm^−1^), glycine (1451 cm^−1^) and alanine (1105 and 917 cm^−1^). Information on the bands identified in the spectrum (Figure 5) is summarized in Table 5.

The most intense band in the spectrum of H-Gly-Hyp-Pro-Ala-Hyp-Pro-OH is at 1451 cm^−1^, assigned to the CH_2_ scissoring vibration. Analyzing wound samples from male ICR mice (at different time points), Liu et al. [69] connected these bands with the total matrix of the wound samples. The area ratio of this band to the amide I band (representing the collagen content) could be used for examining collagen levels in wound and determining the degree of wound healing.

**Figure 6:**
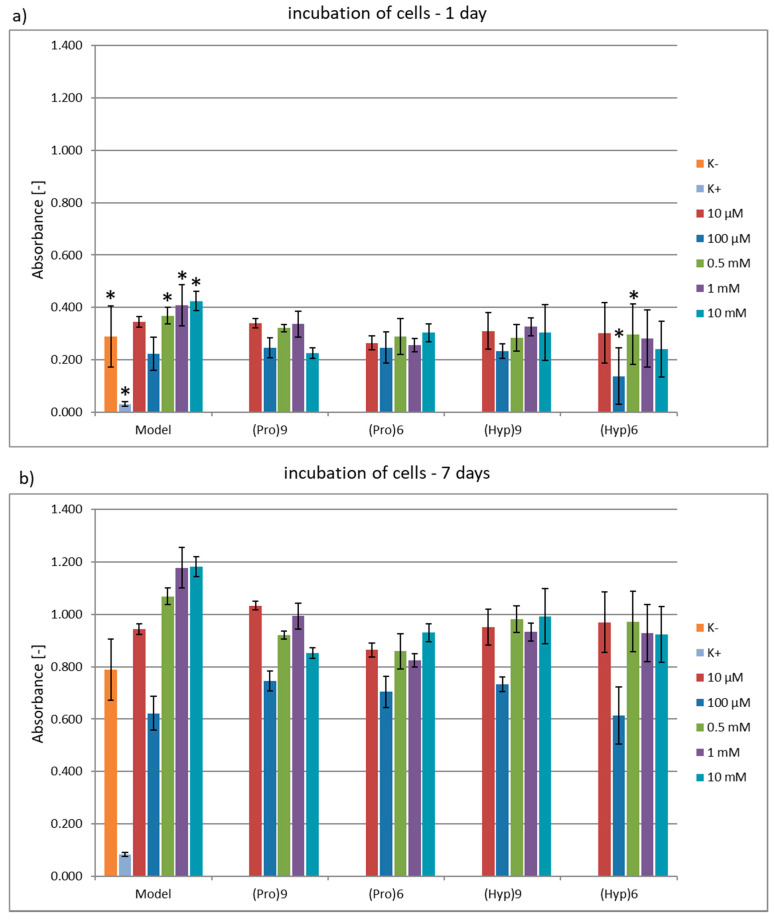
Results of cell metabolic activity by using 3-(4,5-dimethylthiazol-2-yl)-2,5-diphenyltetrazolium bromide (MTT test) of the endothelial cell line EA.h 926 cultured in the presence of peptides **1**–**5**, panel (**a**)—cells incubated in the presence of peptides for 1 day, panel (**b**)—cells incubated in the presence of peptides for 7 days. Cytotoxicity tests were performed in five replicates. The presented results are average values. Statistical significance was assessed using one-way ANOVA analysis of variance. Values for which the value **p < 0.001, ** *p* < 0.01, *p < 0.05 were considered statistically significant (see Supplementary Materials, Figure S1). No statistical significance was observed for 7 days incubation.

All the peptides were also assessed in terms of their cytotoxicity against the endothelial cell line EA.h 926. No cytotoxicity was observed (Figure 6) both for incubating cells in the presence of peptides **1**–**5** for 1 day (Figure 6a) and for 7 days (Figure 6b). Cells cultured in medium without the addition of any substances or harmful agents were used as the Control^−^ (K^−^), while cells treated with DMSO on the second day of incubation were used as the Control^+^ (K^+^). The dimethyl sulfoxide (DMSO) was added to a final concentration of 5%. An MTT test was used to assess the cytotoxicity of peptides **1**–**5**. No cytotoxic effect was observed in a wide range of tested concentrations for any of the used peptides—model peptide **1** which is a fragment of collagen H-Gly-Hyp-Pro-Ala-Hyp-Pro-OH, oligoproline derivatives H-(Pro)_6_-OH (**2**), H-(Pro)_9_-OH (**3**) and oligohydroxyproline derivatives H-(Hyp)_6_-OH (**4**), H-(Hyp)_9_-OH (**5**). The measured absorbance was higher or at least comparable to the results obtained under Control^–^ conditions. The tests were performed in the concentration range of 10 μM and 10 mM.

The highest cytotoxicity for peptides **1**–**5** was observed at 100 μM. However, for subsequent higher concentrations of 0.5 mM and 1 mM all tested peptides showed a reduction in cytotoxicity. At the highest tested concentration of 10 mM, only the H-(Pro)_9_-OH (**3**) peptide showed comparable toxicity to that at 100 μM. For the remaining peptides, no significant cytotoxicity was observed even at the highest concentration. The relationship between cytotoxicity and the concentration of peptides may seem surprising, since the highest cytotoxicity was observed at one of the lower concentrations, this observation may indicate a differentiation of their interaction with different receptors depending on the concentration. The receptors that collagen affects are—integrins, discoidin domain receptors DDR1 and 2, OSCAR, GPVI, G6b-B and LAIR-1 of the leukocyte receptor complex (LRC) and mannose family receptor uPARAP/Endo180 [70].

In the next stage of our research, we attempted to use structurally stable oligoprolines and oligohydroxyprolines, containing the key spatial structure for collagen of proline and hydroxyproline residues, to obtain conjugates with polysaccharides. Nonwovens from chitosan and nonwovens from calcium alginate were used in the tests. The choice of both polysaccharides was dictated by their wide use in medicine, including regenerative medicine. DMT/NMM/TosO^−^ was used to covalently attach peptides **2**–**5** to the polysaccharide matrices. The choice of this reagent was based on its high efficiency for both classical peptide synthesis and ester synthesis in solution and on solid phase, as well as its usefulness for the functionalization of solid surfaces. DMT/NMM/TosO^−^ has been used successfully to functionalize cellulose, alginate, chitosan and carbon nanomaterials [51,71,72,73]. In addition to efficient acylation of nucleophilic groups in the presence of superactive triazine carboxylic acid esters, triazine coupling reagents have one more important property, crucial from the perspective of the modification of solid materials. 1-Hydroxy-4,6-dimethoxy-1,3,5-triazine formed as a side product is easily removed by extraction, which eliminates the adverse process leading to deposits on materials.

**Figure 7:**
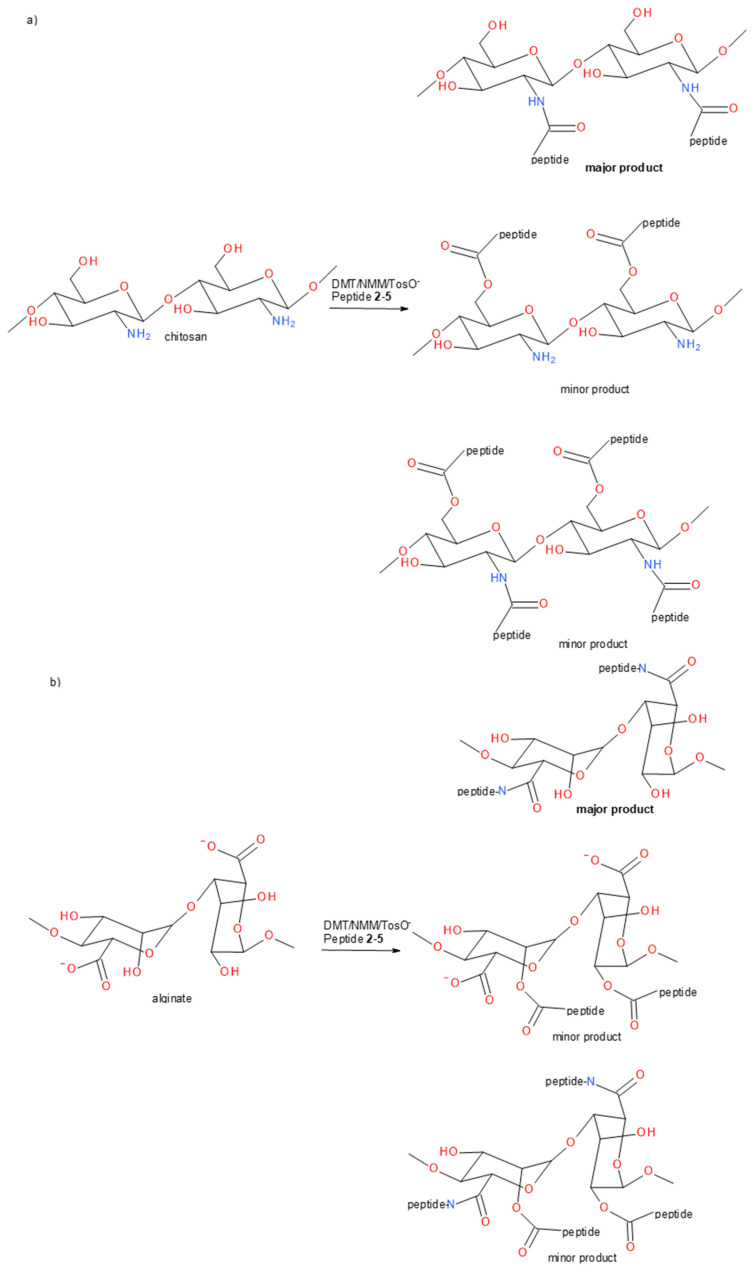
Scheme of the reaction attaching peptides **2**–**5** to chitosan (**a** panel) and calcium alginate (**b** panel) functional groups.

It was assumed that only functional groups on the surfaces of the polysaccharide nonwovens would be modified. Therefore, much smaller amounts of peptides **2**–**5** and the condensing reagents were used relative to the polysaccharide functional groups. About 10 mol.% of the reagents was used for the amino groups of chitosan and carboxyl/hydroxyl alginate. For the acylation of chitosan, it is theoretically possible to acylate amino and hydroxyl groups in a reaction with superactive triazine esters of peptides **2**–**5**. However, due to the significantly higher reactivity of the amine functions, it can be assumed that as a result of modification conjugates are predominantly formed in which the peptide is attached to the chitosan matrix by means of an amide bond (Figure 7a). It is also possible that derivatives are formed linked by ester bonds. A different method of functionalization occurs with calcium alginate. In this case, the hydroxyl groups of the M and G units can be acylated, which leads to polysaccharide-peptide conjugates linked by an ester bond. The presence of carboxyl groups in calcium alginate also allows reactions using carboxyl groups. Under conditions that allow exchange within the carboxylate anion (the release of Ca^2+^ and the formation of either the sodium or ammonium salt in the presence of an amine), there is the possibility to activate the carboxyl function of alginate using triazine condensation reagents, forming superactive alginate triazine esters, followed by aminolysis with amino peptide groups. Under the experimental conditions used (no preactivation of peptides, excess condensation reagent and tertiary amine in relation to the amount of peptides **2**–**5**), it is possible to modify the alginate matrix in two ways (Figure 7b).

**Figure 8:**
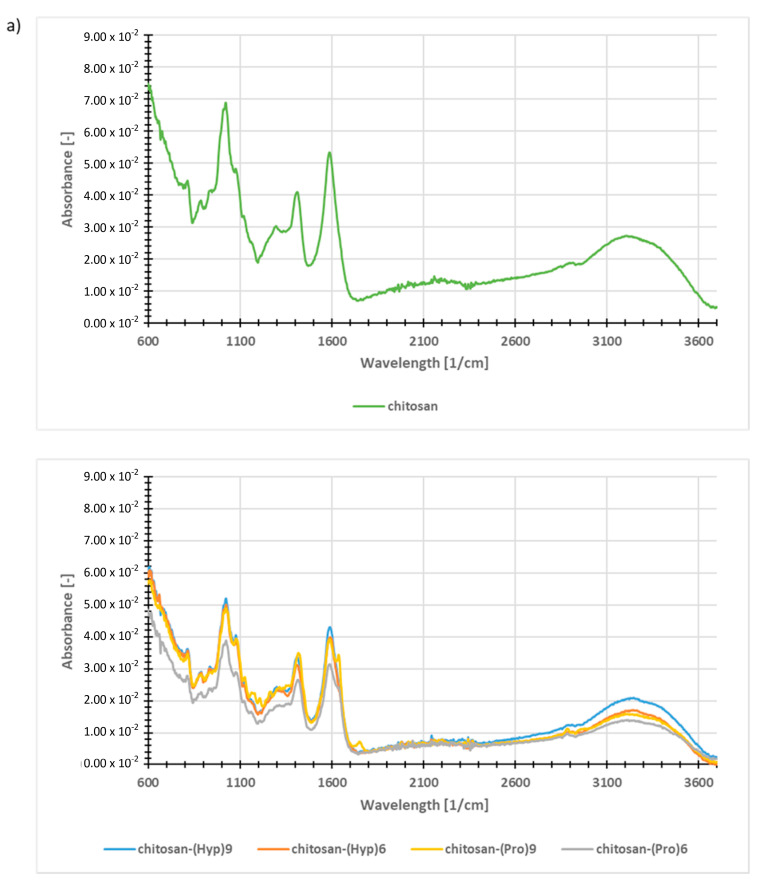

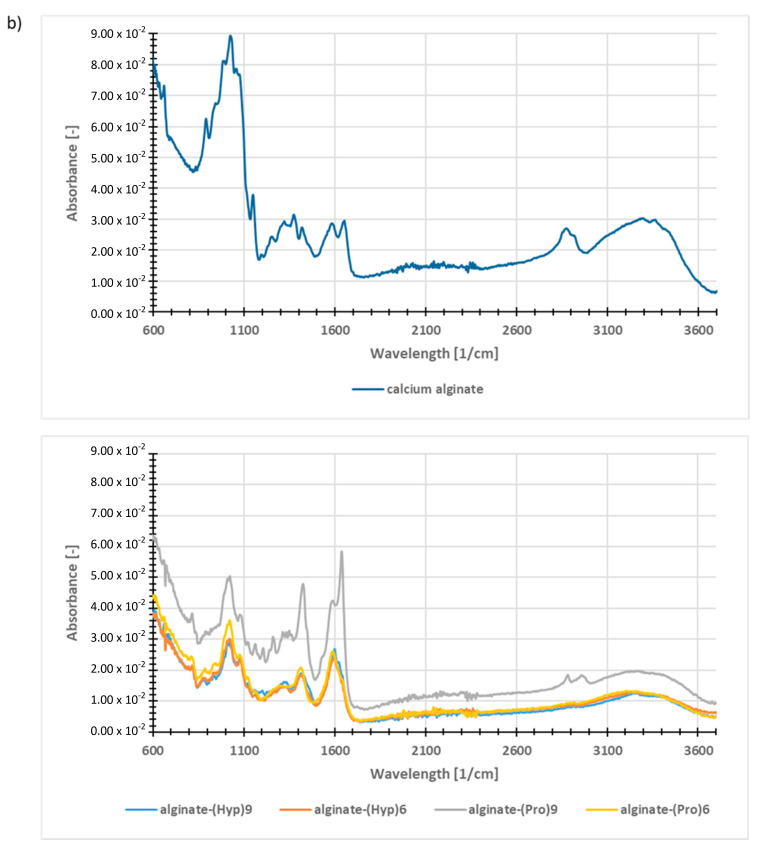
Fourier transform infrared (FT-IR) spectra of polysaccharide-peptide conjugates: (**a**) top panel–unmodified chitosan, bottom panel—chitosan conjugates with peptides **2**–**5**; (**b**) top panel—unmodified calcium alginate, bottom panel—alginate conjugates with peptides **2**–**5**.

The successful attachment of peptides **2**–**5** to alginate and chitosan nonwoven was confirmed by FT-IR analysis (Figure 8). The spectra for the polysaccharide-peptide conjugates were compared to those for the unmodified polysaccharides. The results confirmed the addition of oligoproline derivatives and oligohydroxyproline to the surface of the nonwovens.

The unmodified chitosan nonwoven (Figure 8a, upper panel) has characteristic bands in the range of 1730–820 cm^−1^, which result from the presence of vibrations described in the literature [74] the C=O group derived from residual N-acetyl groups at 1590 cm^−1^; the -CH_3_ group derived from residual N-acetyl groups in the 1400 cm^−1^ range; sugar ring vibrations C-C and glycosidic bond (C-O-C) in the range of 1280–910 cm^−1^. The band observed in the 3293–3362 cm^−1^ range corresponds to N-H and O-H stretching vibrations, as well as to intramolecular hydrogen bonds. In turn, the bands at about 2870 and 2920 cm^−1^ can be attributed to C-H symmetrical and asymmetrical stretching (CH_2_ and CH_3_ groups, respectively). These correspond to bands characteristic of the polysaccharide (skeletal vibrations of the ring). In the case of chitosan nonwoven modified with peptides **2**–**5**, peptide bond bands are visible which overlap partly with the chitosan (Figure 8a, bottom panel). The strongest greatest change in the band relationship compared to the unmodified nonwoven is in the range of 1750–920 cm^−1^. The different band is that for the carbonyl group (1680 cm^−1^), which is derived from the peptide bond. Additional bands in this range are responsible for the stretching vibrations of N-acetyl groups and C-O bonds, at 1580 and 1025 cm^−1^ respectively. The spectrum also shows bands derived from the sugar backbone and the glycosidic bonds in the chitosan chain (1180 cm^−1^, 1040 cm^−1^, 1030 cm^−1^).

Analysis of the spectrum of unmodified calcium alginate (Figure 8b, upper panel) reveals the presence of a characteristic broad band in the range 3600–3000 cm^−1^, characteristic for the stretching vibrations of OH groups. Additionally, two absorption bands corresponding to the valence vibrations of the C-O group of the carboxyl ion (asymmetrical at 1420 cm^−1^ and symmetrical at 1620 cm^−1^) were found. The band with the highest intensity at 1080–950 cm^−1^ is characteristic for the skeletal vibrations of the alginate ring and indicates the presence of a glycosidic bond typical of polysaccharide materials. Analyzing the spectra of calcium alginate conjugates with peptides **2**–**5**, it is possible to identify amine groups shown by stretching vibrations in the range of 3550–3200 cm^−1^ (secondary amines) and by a small peak at 1250 cm^−1^ (Figure 8b, bottom panel). The presence of vibrations in the 3300 cm^−1^ range indicates the presence of carboxyl groups in the peptides. In addition, the bands at 1650–1500 cm^−1^ confirm the presence of peptide bonds. Vibrations from methylene groups are visible in the area of 1410–1500 cm^−1^. The spectra also show a broad band derived from the alginate hydroxyl groups, which unfortunately overlaps with a peptide bond band. The intense band at 1400 cm^−1^ indicates the presence of O-H and C-O bonds. The band in the range of 1150–800 cm^−1^ is derived from the original alginate substrate.

Studying the electrokinetic properties of textiles and nonwovens is very useful, as it allows changes in the charge on the materials surface to be monitored after each step of modification. The electrokinetic potential is the component of the total potential in the intermediate surface layer at the solid/liquid interface. Zeta potential gives information on the nature and dissociation of functional groups, as well as on the hydrophilicity or hydrophobicity of the fiber surface and the absorption of ions and other compounds. The most important value is the isoelectric point (IEP), the pH value for which the electrokinetic surface potential is equal to zero.

**Figure 9:**
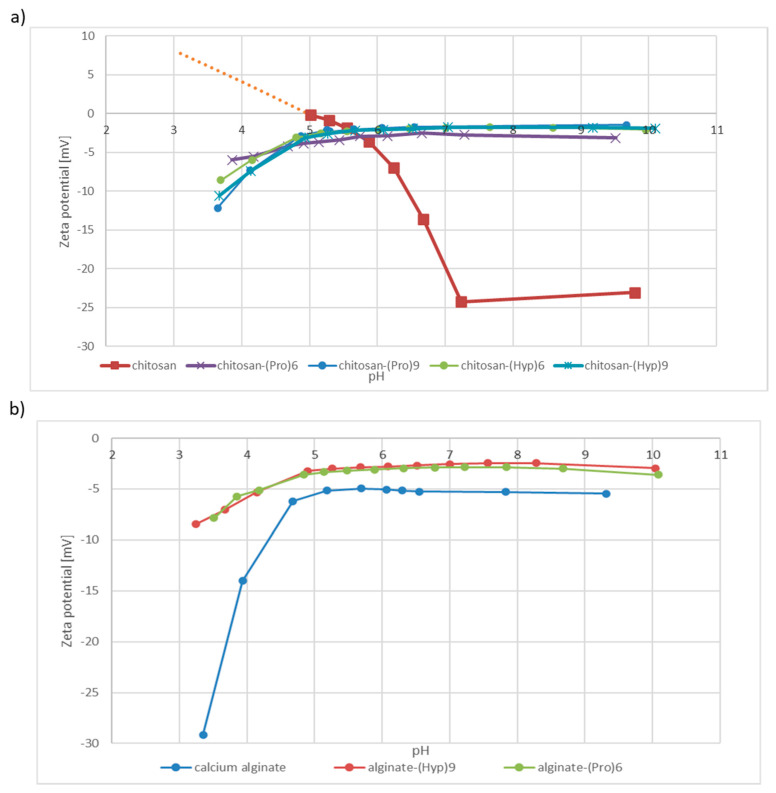
Zeta potential curves for (**a**) chitosan-based materials and (**b**) alginate based materials.

Measurements were performed on the fibers, nonwovens, powders and granules according to the streaming potential method, using a SurPASS electrokinetic analyzer by Anton Paar equipped with a cylindrical measuring cell. The zeta potential of the chitosan and alginate-based materials was determined in terms of the dependence of 0.1 M KCl solution to pH. To adjust the pH, we used 0.1 M sodium hydroxide and 0.1 M hydrochloric acid. The zeta potential measurements show very clearly differences in terms of surface structure between the samples. Lower zeta potential values were observed for unmodified materials compared to samples with peptides **2**–**5** attached to the surface of polysaccharides matrices. At the plateau value of ζ‒potential at pH 8–10, the acidic groups were completely dissociated. The unmodified chitosan has a hydroxyl group on its surface, which was stable in the tested pH range and did not protonate or deprotonate. However, in the presence of free amino groups ammonium salts form in solutions with low pH, which resulted in the formation of a positive charge on the surface of the nonwoven fabric. Since the ζ‒potential is −4 mV, it can be concluded that these amino groups predominated. After the attachment of proline/hydroxyproline derivatives to chitosan, the surface is more positive charged as a result of the formation of an amide bond between chitosan and the carboxyl group. However, the presence of a net charge on a modified chitosan surface may be associated with the protonation of the N-terminal amino group in the attached peptide. In the case of acylation of hydroxyl groups of chitosan by means of superactive triazine esters of peptides **2**–**5**, it is possible to observe the presence of a positive charge on the amine functions of chitosan and the N-terminal amino acid of peptides **2**–**5** in the curves imaging zeta potential. The curves presented in Figure 9a confirm the effective binding of oligoproline and oligohydroxyproline derivatives to the nonwoven surfaces by amide bonds, although the formation of an ester bond cannot be ruled out. Another phenomenon can be observed for all treated surfaces. At pH 4, the surface became more negative due to the shift the shear plane into the liquid phase, causing a reduction of in the ζ-potential [75,76,77]. This indicates fiber swelling.

At the plateau value of ζ‒potential at pH 8–10, the acidic groups are completely dissociated [75,76,77,78,79,80].

The hydroxyproline and proline derivatives were also effective when attached to calcium alginate materials. The unmodified calcium alginate nonwoven showed sorption properties, as reflected in the specific appearance of the zeta potential curve. Alginate fibers bind significant amounts of water, due to their tendency to form a so-called “egg box” structure [78,79,80,81,82]. The penetration into the alginate of water molecules leads to swelling, which has a direct influence on the zeta potential. This may be noticed in Figure 9b, where the zeta potential curve for calcium alginate shows a characteristic increase in negative zeta potential below pH 5. The pH titration curve shows a rather small negative zeta potential (−5 mV) at the plateau value of ζ-potential, rapidly turning to a more negative potential below pH 4, reaching −29.1 mV at pH 3.3. This small negative zeta potential results from the presence of acidic carboxyl and hydroxyl groups on the surface of the alginate fibers, making swelling possible. The curves for Ca‒alginate indicate that, once the water adsorption process has finished and the material is already swollen, the electrochemical double layer transforms into a solid and the shear plane transforms into an electrolyte solution. Similarly to the case of the modified chitosan surface, after modification of the calcium alginate surface using proline/hydroxyproline derivatives higher zeta potential was noticed (‒3.5 mV). The swelling of fibers at the surface is superimposed on the dissociation of surface functional groups above pH 5. Acidic surface groups are introduced, increasing the hydrophilic character of the surface [75‒80]. A difference between the Ca‒alginate before and after modification can be noticed in the pH range of 3‒4. The slope of the curves is not as sharp for the modified surfaces as it is for the untreated Ca-alginate. It can be assumed that after modification of calcium alginate surfaces using proline/hydroxyproline chains the tendency for swelling is limited. The curves obtained for peptide‒alginate conjugates show higher zeta potential and have a similar shape, reflecting the stable physicochemical properties of the final products in the studied pH range.

**Figure 10:**
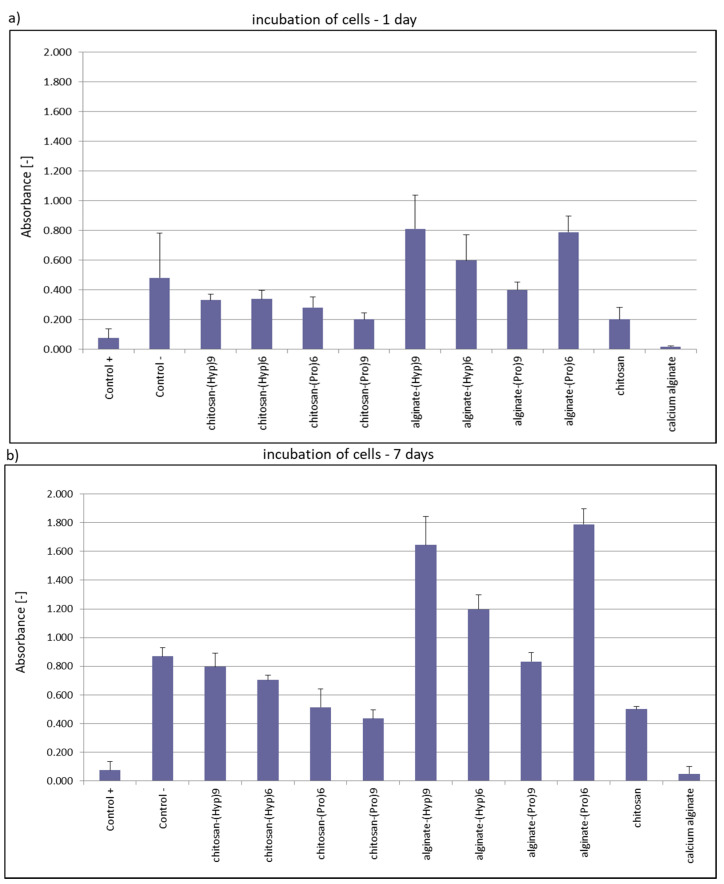
Cytotoxicity of polysaccharide conjugates with **2**‒**5** peptides to the endothelial cell line EA.hy 926, according an MTT assay, panel (**a**)—cells incubated in the presence of peptides for 1 day, panel (**b**)—cells incubated in the presence of peptides for 7 days. Cytotoxicity tests were performed in five replicates. The presented results are average values. No statistical significance was observed for 1 day and 7 days incubation.

In the final stage of the research, we studied the effects of covalently linked conjugates of peptides **2**‒**5** with chitosan and alginate (Figure 10) on the endothelial cell line EA.hy 926. Experiments were performed for incubation of cells for 1 day (Figure 10a) and 7 days (Figure 10b) to check the effect of alginate and chitosan conjugates with peptides **2**‒**5** both in the short and long term, which is important from the point of view of the usefulness of materials used in regenerative medicine. As the Control^‒^ (K^‒^) were again used cells cultured in the medium without the addition of any substances or any harmful factors, while as the Control^+^ (K^+^) were used cell cultures killed by adding DMSO on the second day of incubation. DMSO was added to give a 5% final concentration in the culture medium. Unmodified chitosan and calcium alginate were also studied. Based on absorbance measured by an MTT test, unmodified chitosan showed a 58% reduction in cell viability compared to the Control^−^ and over 2.5‒fold increases in cell viability compared to the Control^+^. A surprising result was observed in the case of unmodified calcium alginate nonwoven. Cell viability relative to the Control^+^ was reduced by more than 75%. The adverse effect of calcium alginate on the endothelial cell line EA.hy 926 may result from the exchange of Ca^2+^ ions from the polysaccharide matrix for monovalent Na^+^ and/or K^+^ ions, causing the release of large amounts of Ca^2+^ ions into the culture medium. This in turn may adversely affect the endothelial cells [83].

The adverse effect of calcium alginate was been completely eliminated by the covalent attachment of **2**‒**5** peptides to the nonwoven fabric. In all cases, a significant increase in absorbance was observed, indicating cell viability. For alginate conjugates with (Hyp)_9_ and (Pro)_6_, the absorbance value was 1.7 times higher compared to the Control^‒^, which indicates that these materials had a very beneficial effect on the cells. A slight decrease in absorbance was observed for the alginate conjugate with (Hyp)_6_, which indicates that in the case of hydroxyproline derivatives a longer peptide chain has a more favorable effect on cell viability. Slightly lower absorbance compared to the Control^‒^ was observed for the alginate conjugate of (Pro)_9_. Surprisingly, a more beneficial effect on cells is observed for shorter peptide derivatives in the case of proline derivatives. Lower absorbance was observed for all conjugates of chitosan with peptides **2**‒**5** compared to the Control^‒^. However, for conjugates with (Hyp)_9_, (Hyp)_6_ and (Pro)_6_ the absorbance value was higher than that obtained for unmodified chitosan. This indicates that the spatially stable oligoproline and oligohydroxyproline derivatives had a positive effect on the endothelial cells. Only in the case of the chitosan‒(Pro)_9_ conjugate was the absorbance value comparable to that for the unmodified polysaccharide, showing no clear positive effect by the proline/hydroxyproline derivatives on cells. In addition, no adverse effect of polysaccharide-peptide conjugates on cells was observed in experiments with extended incubation time (Figure 10b). The most positive effect has been observed for conjugates of alginate with oligoprolines and oligohydroxyprolines. The diversity of biological responses between nonwovens based on polysaccharides may result from their the physico-chemical properties, including their size of the fibers, because the influence of particle size on biological activity is well known [84].

**Figure 11:**
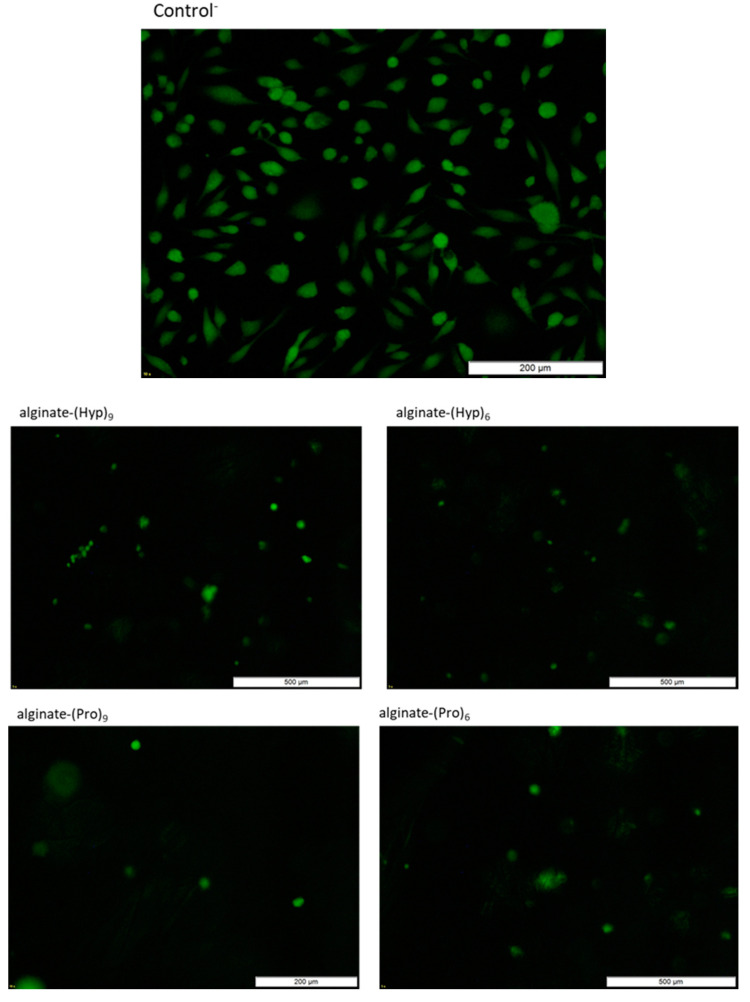

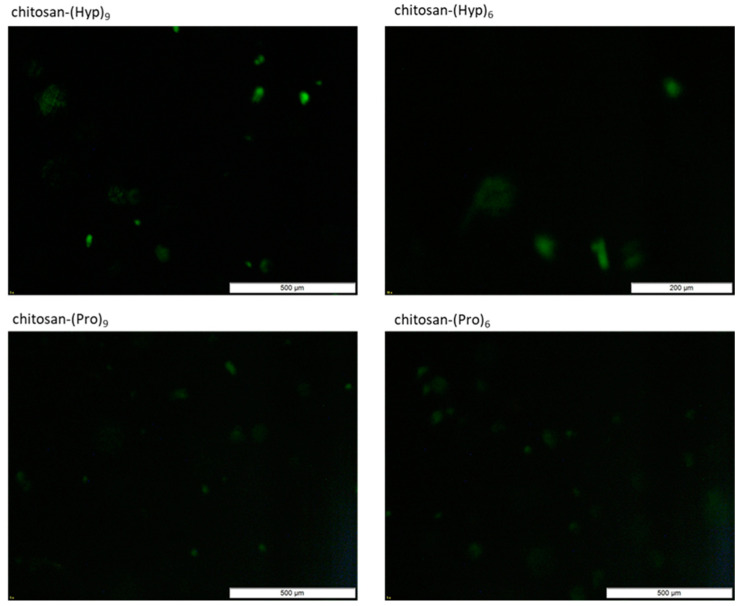
Pictures of the endothelial cell line EA.hy 926 stained with two fluorescent dyes: Calcein AM (green color for live cells) and ethidium homodimer-1 (EthD-1, stains dead cells red).

Live/Dead Viability/Cytotoxicity tests using Calcein AM (a green color for live cells) and ethidium homodimer-1 (EthD-1, a red color for dead cells) also showed a positive effect of alginate and chitosan conjugates with **2**‒**5** peptides on the endothelial cell line EA.hy 926 (Figure 11). However, direct counting of alive and dead cells cultured in the presence of modified chitosan and alginate nonwovens was made difficult by the “melting” of cells in the polysaccharide matrices.

In the case of Control^−^, cells were incubated without any additions hindering the documentation of tests by microscopic technique. In contrast, material swelling and final formation of multidimensional structures (MD) in the form of similar to gels, for which microscopic imaging was difficult, was observed in studies by using polysaccharide nonwovens modified with **2**‒**5** peptides. This difficulty may also have been the result of strong cell binding to the polysaccharide matrix.

The literature describes many examples of peptides derived from collagen, gelatin, elastin, fibronectin and laminins containing the RGD motif, which is considered the most important element improving the activity of various biomaterials, because of the interaction with integrins (pro-adhesive motif). Also, another collagen fragment, laminins that do not contain the RGD motif, affecting cell adhesion. Their characteristic feature is the ability to bind to integrin receptors, which affects in improved adhesion and proliferation of many cells. However, when thinking about the regeneration process, one should not forget that it is a complex process that should be stimulated in many ways. Therefore, derivatives derived from growth factors affecting endothelial cell proliferation, migration, viability and angiogenesis are also useful in medicine. In addition to peptides derived from proteins and polypeptides present in ECM, synthetic peptides belonging to the group of self-assembly peptides (SAPs) are also known, which are also increasingly used in regenerative medicine [85,86,87]. The described oligoproline/oligohydroxyproline derivatives, although belonging to the group of synthetic peptides are able to mimic the collagen spatial structure and positively affect endothelial cell line. In the case of proline derivatives, inhibition of proliferation and cytotoxicity against various cell types is not observed. An additional feature of oligoproline is self-organization in contact with cell membranes, which ultimately results in the ability to penetrate cell membranes [57].

In addition, proline derivatives are characterized by high resistance to hydrolysis in the presence of proteolytic enzymes, which allows their use in materials for regeneration that require prolonged contact with tissues.

However, probably the most important advantage of using oligoprolines/oligohydroxyprolines, which are made of identical amino acids, is the possibility of using the well-known method of synthesis of homopolypeptides by using N-carboxy anhydride (Leuch’s anhydride) procedure that allows the synthesis of longer derivatives on an enlarged scale in solution.

## 4. Conclusions

In this study, we have shown that oligoproline and oligohydroxyproline derivatives (peptides **2**‒**5**) can be used successfully as functional mimetics of collagen, which is the main protein component of the extracellular matrix. Functional similarity was found based on both CD and Raman spectroscopy. In addition, peptides **2**‒**5** were found to have similar biological activity towards the endothelial cell line EA.hy 926 to the model collagen fragment (H-Gly-Hyp-Pro-Ala-Hyp-Pro-OH, **1**). Peptides **2**‒**5** were used to synthesize their conjugates with chitosan and calcium alginate, which are polysaccharides widely used in medicine. To attach the oligoproline and oligohydroxyproline derivatives to the polysaccharide functional groups, 4-(4,6-dimethoxy-1,3,5-triazin-2-yl)-4-methylmorpholinium toluene-4-sulfonate (DMT/NMM/TosO^−^) was used as a condensing reagent. The efficiency of peptide attachment to the polysaccharides was confirmed using the FT-IR method, which showed additional bands characteristic of newly-formed amide and ester bonds. Zeta potential measurements (electrokinetic properties) revealed very clear differences in surface structure between the polysaccharides modified with peptides **2**‒**5** and unmodified carbohydrate matrices. Biological studies (the MTT test and Live/Dead assay) proved that the obtained peptide-polysaccharide conjugates are not toxic to the endothelial cell line EA.hy 926 and in many cases have a highly positive effect on cell proliferation. The results of this study provide the basis for further research on the use of conjugates composed of functional mimetic collagen with polysaccharides in regenerative medicine.

## Figures and Tables

**Table 1:** Parameters for obtaining of conjugates of chitosan with peptides **2**–**5**.

Peptide	Modification of Chitosan Nonwoven	Conjugates
H-(Pro)_6_-OH (**2**)	H-(Pro)_6_-COO-4,6-dimethoxy-1,3,5-triazine ester C=0.01 M in DMF–water (7:3, *v*:*v*), 10% relative to amino groups of D-glucosamine units	Chitosan-(Pro)_6_
H-(Pro)_9_-OH (**3**)	H-(Pro)_9_-COO-4,6-dimethoxy-1,3,5-triazine ester C=0.01 M in DMF–water (7:3, *v*:*v*), 10% relative to amino groups of D-glucosamine units	Chitosan-(Pro)_9_
H-(Hyp)_6_-OH (**4**)	H-(Hyp)_6_-COO-4,6-dimethoxy-1,3,5-triazine ester C=0.01 M in DMF–water (7:3, *v*:*v*), 10% relative to amino groups of D-glucosamine units	Chitosan-(Hyp)_6_
H-(Hyp)**9**-OH (**5**)	H-(Hyp)_9_-COO-4,6-dimethoxy-1,3,5-triazine ester C=0.01 M in DMF–water (7:3, *v*:*v*), 10% relative to amino groups of D-glucosamine units	Chitosan-(Hyp)_9_

**Table 2:** Parameters for obtaining of conjugates of calcium alginate with peptides **2**–**5**.

Peptide	Modification of Chitosan Nonwoven	Conjugates
H-(Pro)_6_-OH (**2**)	Mixture of **2** (1 equiv.) and DMT/NMM/TosO^−^ (3 equiv.) in DMF–water (7:3, *v*:*v*), 10% peptide relative to reactive groups of MM, GG or MG/GM units	Alginate-(Pro)_6_
H-(Pro)_9_-OH (**3**)	Mixture of **3** (1 equiv.) and DMT/NMM/TosO^−^ (3 equiv.) in DMF–water (7:3, *v*:*v*), 10% peptide relative to reactive groups of MM, GG or MG/GM units	Alginate-(Pro)_9_
H-(Hyp)_6_-OH (**4**)	Mixture of **4** (1 equiv.) and DMT/NMM/TosO^−^ (3 equiv.) in DMF–water (7:3, *v*:*v*), 10% peptide relative to reactive groups of MM, GG or MG/GM units	Alginate-(Hyp)_6_
H-(Hyp)**9**-OH (**5**)	Mixture of **5** (1 equiv.) and DMT/NMM/TosO^−^ (3 equiv.) in DMF–water (7:3, *v*:*v*), 10% peptide relative to reactive groups of MM, GG or MG/GM units	Alginate-(Hyp)_9_

**Table 3:** Band assignments for Raman spectra of (Pro)_9_ and (Pro)_6_ peptide structures based on [63,65,66,67,68].

Peak Position (cm^−1^)		Proposed Band Assignment
(Pro)_9_	(Pro)_6_	
1736	1738	C=O stretch
1660	1660	Amide I (C=O stretch)
1616	1614	C=O asymmetric stretch
1451	1453	CH_2_ scissor or CH_2_ bend
1272	1274	Amide III (C-N stretch and N-H bend)
1245	1245	C-O stretch
1197	1197	NH^+^ deformation
1165	1165	NH_2_^+^ deformation
1096	1096	CH_2_ rock
1050	1051	C-N stretch
974	974	C-C-N stretch
923	924	C-COO^−^ stretch
836	836	C-C stretch or CH_2_ rock
730	731	COO^−^ deformation
417	417	OCC bend–2011 Kecel
365	367	NCC bend–2011 Kecel

**Table 4:** Band assignments for Raman spectra of (Hyp)_9_ and (Hyp)_6_.

Peak Position (cm^−1^)		Proposed Band Assignment
(Hyp)_9_	(Hyp)_6_	
1734	1730	C=O stretch
1660	1660	Amide I (C=O stretch)
1642	1642	COO^−^ asymmetric stretch
1611	1611	C=O asymmetric stretch
1465	1463	CH_2_ bend or CH_2_ scissor
1445	1445	CH_2_ bend
1344	1344	C-H bend or CH_2_ twist
1313	1320	C-H bend
1267	1267	Amide III (C-N stretch and N-H bend)
1235	1232	C-O stretch
1165	1165	NH_2_^+^ deformation
1085	1085	CH_2_ rock
971	974	C-C-N stretch
917	917	C-COO^−^ stretching
882	882	NH_2_^+^ rock, N-H bend
829	825	C-C stretch or CH_2_ rock
727	727	COO^−^ deformation
560	558	skeletal deformations
336	329	CCN bend
286	288	skeletal deformations

**Table 5:** Band assignments for Raman spectra of H-Gly-Hyp-Pro-Ala-Hyp-Pro-OH [63,64,65,66,67].

Peak Position (cm^−1^)	Proposed Band Assignment	Proposed Residue
1740	C=O stretch	Pro/Hyp
1662	Amide I (C=O stretch)	–
1613	C=O asymmetric stretch	Pro/Hyp
1451	CH_2_ scissor or CH_2_ bend	Pro/Gly
1344	C-H bend or CH_2_ twist	Pro/Hyp
1307	COH bend	Pro
1269	Amide III (C-N stretch and N-H bend)	–
1243	C-O stretch	Pro/Hyp
1197	NH^+^ deformation	Pro
1105	C-C stretch	Ala
1057	C-N stretch	Pro
976	C-C-N stretch	Pro/Hyp
917	C-COO^−^ stretch	Pro/Hyp/Ala
880	NH_2_^+^ rock, N-H bend	Pro/Hyp
836	C-C stretch	Pro/Hyp
818	C-C stretch	Pro/Hyp
742	–	Pro
724	COO- deformation	Backbone
339	CCN bend	Pro/Hyp
266	skeletal deformations	–
192	CCC bend	–
142	CCC bend	–

## Supplementary Materials

The following are available online at https://www.mdpi.com/1996-1944/13/14/3079/s1, Figure S1: Results of MTT test of the endothelial cell line EA.h 926 cultured in the presence of peptides 1–5 (1 day of incubation) – Statistical analysis.
