# Supplementary material for: Conjugates of Chitosan and Calcium Alginate with Oligoproline and Oligohydroxyproline Derivatives for Potential Use in Regenerative Medicine
Source: Materials (Basel). 2020 Jul 10;13(14):3079. doi: 10.3390/ma13143079 (PMC7412561; doi:10.3390/ma13143079)
Supplement: Supplementary file 1 [file materials-13-03079-s001.pdf]

materials

MDPI

Supplementary Materials

Conjugates of Chitosan and Calcium Alginate with Oligoproline and Oligohydroxyproline Derivatives for Potential Use in Regenerative Medicine

Model pep, 1	10 µm	100 µm	0.5 mM	1 mM	10 mM	K-	K+
10 µm	-	ns	ns	ns	ns	ns	*
100 µm		-	*	*	*	ns	*
0.5 mM			-	ns	ns	ns	*
1 mM				-	ns	*	*
10 mM					-	*	*
K-						-	*
K+							-

(Pro)9, 3	10 µm	100 µm	0.5 mM	1 mM	10 mM	K-	K+
10 µm	-	ns	ns	ns	ns	ns	*
100 µm		-	ns	ns	ns	ns	*
0.5 mM			-	ns	ns	ns	*
1 mM				-	ns	ns	*
10 mM					-	ns	*
K-						-	*
K+							-

(Pro)6, 2	10 µm	100 µm	0.5 mM	1 mM	10 mM	K-	K+
10 µm	-	ns	ns	ns	ns	ns	*
100 µm		-	ns	ns	ns	ns	*
0.5 mM			-	ns	ns	ns	*
1 mM				-	ns	ns	*
10 mM					-	ns	*
K-						-	*
K+							-

(Hyp)9, 5	10 µm	100 µm	0.5 mM	1 mM	10 mM	K-	K+
10 µm	-	ns	ns	ns	ns	ns	*
100 µm		-	ns	ns	ns	ns	*
0.5 mM			-	ns	ns	ns	*
1 mM				-	ns	ns	*
10 mM					-	ns	*
K-						-	*
K+							-

Figure S1. Results of MTT test of the endothelial cell line EA.h 926 cultured in the presence of peptides **1–5** (1 day of incubation) – Statistical analysis. Statistical significance was assessed using one-way

ANOVA analysis of variance. Values for which the value **$p < 0.001$, ** $p < 0.01$, *$p < 0.05$ were considered statistically significant.
